# Randomized prospective phase III trial of ^68^Ga-PSMA-11 PET/CT molecular imaging for prostate cancer salvage radiotherapy planning [PSMA-SRT]

**DOI:** 10.1186/s12885-018-5200-1

**Published:** 2019-01-07

**Authors:** Jeremie Calais, Johannes Czernin, Wolfgang P. Fendler, David Elashoff, Nicholas Nicholas G. Nickols

**Affiliations:** 1 Department of Molecular & Medical Pharmacology, Ahmanson Translational Theranostics/ Imaging Division, University of California, Los Angeles, USA; 2 Department of Nuclear Medicine, University Hospital Essen, Essen, Germany; 3 Department of Medicine Statistics Core (DOMStat), UCLA CTSI Biostatistics and Computational Biology, University of California, Los Angeles, USA; 4 Department of Radiation Oncology, University of California, Los Angeles, USA; 5 Department of Radiation Oncology, VA Greater Los Angeles Healthcare System, Los Angeles, California USA

**Keywords:** Prostate cancer, PSMA, PET/CT, Randomized phase 3 trial, Salvage radiation therapy

## Abstract

**Background:**

Salvage radiotherapy (SRT) for prostate cancer (PCa) recurrence after prostatectomy offers long-term biochemical control in about 50–60% of patients. SRT is commonly initiated in patients with serum PSA levels < 1 ng/mL, a threshold at which standard-of-care imaging is insensitive for detecting recurrence. As such, SRT target volumes are usually drawn in the absence of radiographically visible disease. ^68^Ga-PSMA-11 (PSMA) PET/CT molecular imaging is highly sensitive and may offer anatomic localization of PCa biochemical recurrence. However, it is unclear if incorporation of PSMA PET/CT imaging into the planning of SRT could improve its likelihood of success. The purpose of this trial is to evaluate the success rate of SRT for recurrence of PCa after prostatectomy with and without planning based on PSMA PET/CT.

**Methods:**

We will randomize 193 patients to proceed with standard SRT (control arm 1, *n* = 90) or undergo a PSMA PET/CT scan (free of charge for patients) prior to SRT planning (investigational arm 2, *n* = 103). The primary endpoint is the success rate of SRT measured as biochemical progression-free survival (BPFS) after initiation of SRT. Biochemical progression is defined by PSA ≥ 0.2 ng/mL and rising. The randomization ratio of 1:1.13 is based on the assumption that approximately 13% of subjects randomized to Arm 2 will not be treated with SRT because of PSMA-positive extra-pelvic metastases. These patients will not be included in the primary endpoint analysis but will still be followed. The choice of treating the prostate bed alone vs prostate bed and pelvic lymph nodes, with or without androgen deprivation therapy (ADT), is selected by the treating radiation oncologist. The radiation oncologist may change the radiation plan depending on the findings of the PSMA PET/CT scan. Any other imaging is allowed for SRT planning in both arms if done per routine care. Patients will be followed until either one of the following conditions occur: 5 years after the date of initiation of randomization, biochemical progression, diagnosis of metastatic disease, initiation of any additional salvage therapy, death.

**Discussion:**

This is the first randomized phase 3 prospective trial designed to determine whether PSMA PET/CT molecular imaging can improve outcomes in patients with PCa early BCR following radical prostatectomy.

**Acronym:**

PSMA-SRT Phase 3 trial.

**Clinical trial registration:**

- ■ IND#130649
  - ◦ Submission: 04.26.2016
  - ◦ Safe-to-proceed letter issued by FDA: 05.25.2016
- ■ UCLA IRB #18–000484,
  - ■ First submission: 3.27.2018
  - ■ Date of approval: 5.31.2018
- ■ UCLA JCCC Short Title NUC MED 18–000484
- ■ NCI Trial Identifier NCI-2018-01518
- ■ ClinicalTrials.gov Identifier NCT03582774
  - ■ First Submitted: 06.19.2018
  - ■ First Submitted that Met QC Criteria: 06.27.2018
  - ■ First Posted: 07.11.2018
  - ■ Last Update Submitted that Met QC Criteria: 07.17.2018
  - ■ Last Update Posted: 07.19.2018

**Trial status:**

Current Trial Status Active as of 08/13/2018

Trial Start Date 09/01/2018-Actual

Primary Completion Date 09/01/2023-Anticipated

Trial Completion Date 09/01/2024-Anticipated

## Introduction

### Background

Prostate cancer (PCa) is expected to have an incidence of 161,000 and a mortality of 27,000 in the US in 2017 [1]. Curative treatments for localized PCa include radical prostatectomy or radiotherapy [2]. After failure of local therapy, recurrence is detected by rising serum PSA levels. Biochemical recurrence (BCR) occurs in 20 to 80% of patients within 10 years after radical prostatectomy, with the risk of failure dependent on NCCN risk group, pathologic features, and genomic classification [3, 4]. Locally recurrent disease after radical prostatectomy may be cured by salvage radiation therapy (SRT) [5]. Overall, SRT offers long-term biochemical control in about 50–60% of patients [6, 7], depending on pre-SRT prostate-specific antigen (PSA) [5], RT dose [8] and risk group [9]. Results from RTOG 0534 trial [10], which compared three salvage RT regimens: SRT directed to the prostate bed alone, SRT directed to the prostate bed with 4–6 months of concurrent ADT, and SRT directed to the prostate bed and nodes with 4–6 months of concurrent ADT, was recently presented [11]. Five-year biochemical recurrence free survivals were higher than expected in all arms at 71.1, 82.7, and 89.1%, respectively. Pre-SRT PSA levels were < 1.0 ng/mL in 90% of patients and only 17% of patients enrolled had a pathologic Gleason Score of 8 or higher. It is likely that these findings will impact routine practice [11]. For high-risk patients, however, 5-year BCR after SRT reaches 70% [6, 12, 13].

Intuitively, SRT is only curative if recurrent disease is completely encompassed by the irradiated volumes. Therefore, accurate localization of recurrent disease is critical. However, standard-of-care imaging modalities are too insensitive to identify recurrence sites in time to guide salvage treatment [14–16]. In practice, SRT is commonly initiated in patients with serum PSA levels < 1 ng/mL, a threshold at which standard-of-care imaging is insensitive for detecting recurrence [15]. As such, SRT target volumes are usually delineated in the absence of radiographically visible disease (gross disease). Based on an expert consensus panel the RTOG (Radiation Therapy Oncology Group) published contouring guidelines for both prostate bed and pelvic lymph node (LN) Clinical Target Volumes (CTV) as these regions often harbor occult tumors not seen on standard-of-care imaging [17, 18]. These consensus CTV are applied in ongoing trials and guide routine clinical care.

The effectiveness of any local therapy depends on accurate imaging to rule out areas of disease that would remain untreated. The lack of sensitivity of standard-of-care imaging for recurrent PCa combined with a sensitive and specific biomarker of early disease recurrence (serum PSA level) generates a unique challenge for local treatment of PCa BCR: cancer is present, but we do not know where it is. There is thus an unmet clinical need to improve target delineation in patients with potentially curable PCa with early BCR.

^68^Ga-PSMA-11 (PSMA) PET/CT is superior to standard-of-care imaging for detecting regional and distant metastatic recurrent PCa at low PSA levels [19–22], highly specific [22] and reproducible [23]. Detection rates of about 50% are reported even at PSA levels of < 0.5 ng/ml [21, 22] and greater than 95% when PSA > 2 ng/mL [24, 21]. PSMA PET/CT outperformed planar bone scan for detection of osseous metastases in large retrospective analyses [25, 26]. The detection rate of PSMA PET/CT for recurrent PCa exceeds that of choline PET/CT [27, 28], and may exceed that of ^18^F-Fluciclovine PET/CT [29].

Therefore PSMA PET/CT has the potential to guide and improve SRT planning in numerous ways [30]. First, PSMA PET/CT defined gross disease within a target volume can be prescribed a higher dose. Second, CTVs can be expanded to encompass areas of disease not seen by current first-line imaging and not normally targeted by consensus CTVs. Third, evidence of metastatic disease indicates that local therapy alone would not offer cure, SRT may be considered futile and abandoned. The potential impact of PSMA PET/CT on SRT planning has been assessed in several studies (Table 1) [30–43]. Taken as a group, the pooled median rate of impact of PSMA PET/CT on SRT planning is 50% (range 17–87%). Our recent multicenter post-hoc analysis of 270 patients with early BCR after prostatectomy showed that PSMA PET/CT would have had a major impact in 19% of patients [38]. Importantly, a major impact was defined as PSMA-positive disease outside planning target volumes expanded from CTVs covering both the prostate bed and pelvic lymph nodes up to L4/L5, which is more expansive than the volumes used in RTOG 0534 [10, 38]. Overall, the addition of PSMA PET/CT would have had an impact on SRT planning in half of patients with a PSA < 1 ng/mL even when using the most generous target volumes. This is the most relevant patient cohort to assess the impact of PSMA PET/CT on SRT.

**Table 1:** Studies That Assessed Impact of PSMA PET/CT on SRT Planning

Author	Year	*n*	PSA (ng/ml) Median (range)	PSMA+	Extrapelvic PSMA+	Any SRT Planning Change	SRT Considered Futile
Shakespeare	2015	18	1.1 (0.017–20.4)	NA	NA	46%	NA
van Leeuwen	2015	70	0.2 (0.05–0.99)	55%	6%	35%	7%
Sterzing	2016	42	2.8 (0.16–113)	60%	NA	61%	NA
Bluemel	2016	45	0.67 (0.10–11.2)	54%	9%	42%	4%
Albisinni	2016	48	2.2 (0.72–6.7)	NA	NA	76%	NA
Schiller	2017	31	0.71 (0.12–14.7)	100%	3%	87%	0%
Henkenberens	2017	39	1.2 (0.3–15.5)	85%	46%	59%	13%
Schmidt-Hegemann	2017	49	0.49 (0.15–6.24)	NA	4%	57%	NA
Habl	2017	83	0.69 (0.09–14.7)	71%	10%	57%	0%
De Bari	2018	12	0.51 (0.10–1.62)	NA	NA	17%	8%
Koerber	2018	71	1.2 (0.03–41.24)	NA	51%	54%	3%
Frenzel	2018	75	0.2 (0.02–653.2)	NA	NA	43%	NA
Farolfi	2018	119	0.32 (0.20–0.50)	35%	21%	30%	18%
Calais	2018	270	0.48 (0.03–1.0)	49%	13%	19%	12%

Few retrospective studies with short-term follow-up reported patient outcome after PSMA PET/CT-based SRT for PCa post-prostatectomy recurrence (Table 2) [44–48]. The mean response rate from these studies is 74% (range 60–83%) after a mean follow-up time of 19 months (range 10.5–29). Interestingly Emmet et al. reported in 99 patients with BCR and PSA 0.05 to 1.0 ng/mL that PSMA PET was independently predictive of treatment response to SRT and stratified men with good response to SRT (negative PSMA (85%) or fossa-confined PSMA (81%)) versus men with poor response to SRT (PSMA N1 (61%) or PSMA M1 (30%)) after a median follow-up of 10.5 months [46].

**Table 2:** Studies That Assessed Outcome of PSMA PET/CT-based SRT

Author	Year	*n*	Response rate	Median follow-up (months)
Henkenberens	2017	23	79%	12.4
Zschaeck	2017	20	60%	29
Emmett	2017	99	72%	10.5
Schmidt-Hegemann	2018	129	83%	20
Schmidt-Hegemann	2018	90	78%	23

However, it remains unclear if incorporation of PSMA PET/CT imaging into the planning of SRT could improve its likelihood of success. There is no randomized prospective trial designed to determine whether PSMA PET/CT can improve outcome at 5 years in patients with PCa early BCR following radical prostatectomy**.** The purpose of this trial is to evaluate the success rate of SRT for recurrence of PCa after prostatectomy with and without planning based on PSMA PET/CT.

### Rationale for study design and hypothesis

**Fig. 1:**
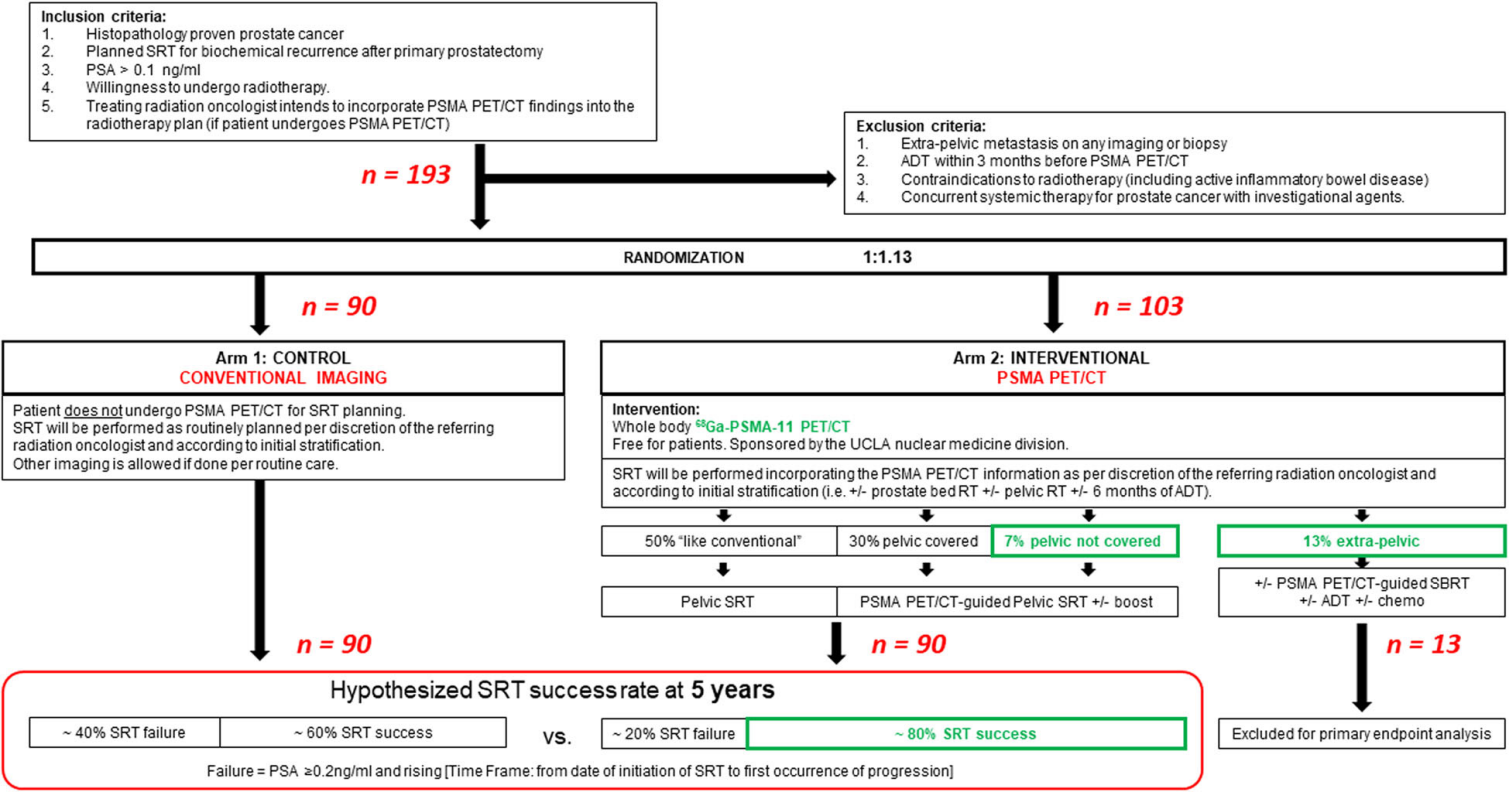
Study Design. 68Ga-: Gallium-68; ADT: Androgen deprivation therapy; PET/CT: Positron Emission tomography/Computed Tomography; PSA: Prostate-specific antigen; PSMA: Prostate-specific membrane antigen; SRT: Salvage Radiation Therapy; SBRT: Stereotactic Body Radiation Therapy; UCLA: University of California, Los Angeles

The overall study design is shown in Fig. 1.

In our recent multicenter post-hoc analysis of 270 patients with early BCR (PSA < 1.0 ng/ml) after prostatectomy we found that 52/270 patients (19%) had at least one lesion detected by PSMA PET/CT which was not covered by the standard radiation fields that covered both the prostate bed and pelvic lymph nodes (RTOG consensus delineations with superior extent extended to L4/L5): extra-pelvic disease was seen in 13% of patients and out-of-field pelvic disease was seen in 7% of patients [38]. SRT delivered based on these volumes would not have generated a durable PSA response. PSMA PET/CT imaging can improve patient selection for successful SRT by excluding patients with M1 disease where SRT would not be curative (13%) and by improving the coverage of the recurrent lesions by the pelvic radiation fields (7%). Therefore, we hypothesized that the incorporation of PSMA PET/CT to SRT planning will improve 5-year PFS by 20%. Based on available published literature [5–9, 12] we assume 5-year PFS to be 60% in Arm 1 (standard SRT) and 80% in Arm 2 (PSMA PET/CT based SRT).

We also assume that approximately 13% of subjects randomized to Arm 2 will have extra-pelvic metastasis detected by PSMA PET/CT, and therefore are not curable by SRT directed to the pelvis [38]. Hence the primary endpoint of the trial is the success rate of SRT. If PSMA PET/CT detects distant metastases, then local salvage with SRT is not a medically indicated therapy because it is not curative. It is therefore not standard-of-care to perform SRT in patients with evidence of distant metastases. The current standard-of-care is to offer SRT to patients who have no evidence of metastatic disease. We acknowledge that patients in the trial who do not undergo PSMA PET/CT may have “hidden/unknown” metastatic disease, but these patients would still receive SRT per routine care. An intention to treat analysis would classify subject who undergo PSMA PET/CT and are found to have distant metastases as failure even without having received treatment. Therefore, the primary endpoint of the trial is the success rate of SRT in patients who actually receive SRT. The question the trial asks is: “*Within patients for whom SRT is appropriate given the imaging results, is the rate of SRT success different between standard-of-care imaging and PSMA PET/CT arms?*”. As such, it would be inappropriate for patients who do not undergo SRT to be included for the primary endpoint. Therefore, patients with PSMA PET/CT showing distant metastases will not be included for the primary endpoint analysis but will still be followed. Based on these estimates, 193 subjects are required to be randomized in a 1:1.13 ratio (90 in control group and 103 in the PSMA group). Randomized, eligible, sample size in each group is therefore *n* = 90.

### Objective of the trial

To evaluate the success rate of salvage radiation therapy (SRT) for recurrence of PCa after prostatectomy with and without planning based on PSMA PET/CT.

### Trial design

Interventional Phase III Randomized Controlled Parallel Assignment Prospective Open Label Clinical Trial.

*Allocation Ratio*: 1:1.13 ratio

*Framework:* Superiority

## Methods

### Study population

Patients with recurrence of PCa after primary radical prostatectomy.

It is anticipated that a total of 193 subjects will be recruited. Such a number is considered appropriate to achieve statistical power for the endpoints of this clinical trial.

*Inclusion criteria*:

1. Histopathology proven PCa
2. Planned SRT for recurrence after primary prostatectomy
3. PSA ≥ 0.1 ng/ml at time of enrollment
4. Willingness to undergo radiotherapy.
5. Treating radiation oncologist intends to incorporate PSMA PET/CT findings into the radiotherapy plan if patient undergoes PSMA PET/CT

*Exclusion criteria*:

1. Extra-pelvic metastasis on any imaging or biopsy
2. Androgen deprivation therapy (ADT) within 3 months before PSMA PET/CT
3. Contraindications to radiotherapy (including active inflammatory bowel disease)
4. Concurrent systemic therapy for PCa with investigational agents.

### Intervention

#### Study procedure

Patients allocated to the PSMA SRT arm (arm 2) will undergo one PSMA PET/CT scan at the UCLA Nuclear Medicine clinic before SRT planning.

#### Investigational PET imaging drug

We will use ^68^Ga-PSMA-11 (Gallium-68-labeled PSMA-ligand Glu-urea-Lys(Ahx)-HBED-CC) as the PET radiopharmaceutical. The administered activity is 3–7 mCi i.v.

#### Source of the study drug

UCLA Biomedical Cyclotron, 780 Westwood Plaza, Los Angeles, CA 90095; Ga-generator generator from Eckert and Ziegler Isotope Products.

#### PET/CT imaging protocol specific

Oral hydration and voiding are recommended immediately before start of the scan. Oral contrast and IV contrast will be administered if not contraindicated. PET/CT images will be obtained using the Siemens Biograph 64 and mCT scanners and will be acquired in 3D mode at 50–100 min after injection of 3–7 mCi of ^68^Ga-PSMA-11. Scan coverage will extend from mid-thigh to the vertex, starting from the mid-thighs. A minimum of 3 min per bed position will be used (weight-based protocol) [49]. Attenuation correction of PET emission data is using segmented CT data. PET images are reconstructed using ordered subset expectation maximization (OSEM) with 2 iterations and 8 subsets.

#### PET/CT imaging analysis

PET/CT Images will be reviewed and analyzed using Siemens Syngo/TrueD and OSIRIX workstations by a board certified nuclear medicine physician and a board certified radiologist experienced in reading PET/CT using recent reporting guidelines (PROMISE criteria, miTNM standardized framework) [50].

#### PET/CT DICOM images transfer

CD/DVD containing the PSMA PET/CT DICOM images and PET/CT report will be delivered to the treating radiation oncologist.

#### Salvage radiation therapy management

The treating physician will be asked to describe their general treatment plan prior to randomization. The choice of treating the prostate bed alone vs prostate bed and pelvic lymph nodes, with or without ADT, is at the discretion of the treating physician.

Patients randomized to control arm 1 do not undergo PSMA PET/CT: SRT will be performed as routinely planned per discretion of the treating radiation oncologist in accordance with the initial general treatment plan whenever possible. Any other imaging is allowed for SRT planning if done per routine care.

Patients randomized to arm 2 will undergo a PSMA PET/CT scan before SRT planning.

- If the PSMA PET/CT scan is negative for BCR site identification: SRT will be performed as routinely planned per discretion of the treating radiation oncologist in accordance with the initial general treatment plan whenever possible. The treating physician is encouraged not to de-escalate therapy as a negative PSMA PET/CT does not mean that the patient has no recurrent PCa, rather that it was unable to be detected by the scan (for example, microscopic disease). Thus, if the initial treatment intent was to treat the prostate bed and pelvic nodes, then this should be pursued even in the absence of PSMA-positive nodes.
- if the PSMA PET/CT scan is positive for pelvic lesions: SRT can be performed with adapted/extended target volumes to include all pelvic PSMA-positive lesions within the radiation fields. SRT may also be performed with focal dose escalation on the PSMA-positive lesions if feasible. SRT can also be performed as routinely planned in accordance with the initial general treatment plan per discretion of the treating radiation oncologist. Furthermore, a PSMA PET/CT scan showing PSMA-positive disease in one or more pelvic nodes does not exclude the possibility of additional disease in the prostate bed, and vice versa.
- If the PSMA PET/CT scan detects PSMA-positive lesions outside the pelvis: Treatment management will be performed as per discretion of the treating radiation oncologist. We assume that approximately 13% of subjects randomized to Arm 2 will have PSMA-positive distant metastases. These patients will not be included in analysis of the primary endpoint, and their actual treatment plan will be determined by the treating radiation oncologist.

### Outcome measures

#### Primary endpoint measure

Success rate of SRT measured as rate of biochemical progression-free survival (BPFS) after initiation of SRT (Time Frame: From date of initiation of SRT until the date of first documented progression or death from any cause, whichever comes first, assessed up to 5 years). Biochemical progression is defined as a serum PSA ≥ 0.2 ng/mL and rising after completion of SRT (second confirmatory value must be rising and separated by ≥ 1 month).

#### Secondary endpoints measures

1. 5-year BPFS rate from date of randomization (Time Frame: 5 years)
2. Metastasis free-survival from date of randomization (Time Frame: 5 years). Diagnosis of extra-pelvic metastatic (M1) disease can be obtained by any imaging or biopsy.
3. Initiation of additional salvage therapy after completion of SRT measured as rate of additional PCa therapy initiation-free survival (Time Frame: 5 years; from the initiation of SRT until the first documented initiation of any additional PCa treatment).
4. Change in initial treatment intent.

### Timeline

#### Screening and enrollment

1. Patients seen for consultation in a radiation oncology, urology, or nuclear medicine clinic who are being evaluated for potential SRT will be informed of this clinical study. The decision to participate will be entirely voluntary. All subjects must sign and personally date the IRB approved informed consent form (ICF) after receiving detailed written and verbal information about the reason, the nature and the possible risks of the trial.
2. UCLA patients will consent after a thorough discussion between the patient and the consenting UCLA physician.
3. All other patients (outside UCLA) will consult over the phone with the UCLA nuclear medicine research team to discuss the study protocol. Signed ICF will be obtained by fax or email.
4. To register and enroll a patient, the UCLA nuclear medicine research coordinator will obtain all required medical documentation, signed informed consent and signed Health Information Portability and Accountability Act (HIPAA) authorization form (by fax or email if patient is from outside UCLA).

#### Randomization and intervention

5) Enrolled patients will be randomized to either the control arm (arm 1) or the PSMA SRT arm (arm 2) in a 1:1.13 ratio. The randomization number and assigned arm will be communicated by phone or email to treating physicians and patients 1 day after the enrollment.
6) Patients assigned to arm 2 will be scheduled to undergo a PSMA PET/CT scan at UCLA Nuclear Medicine free of charge prior to radiation therapy planning. DICOM images and reports of PSMA PET/CT scans will be delivered to the treating radiation oncologist.
7) SRT will be done per routine care at the treating radiation oncologist institution, and typically takes place over about 2 months. The treating radiation oncologist may change the radiation plan depending on the findings of the PSMA PET/CT scan.

#### Follow-up

Current standard-of-care includes weekly on treatment visits during radiotherapy followed by follow-up visits with radiation oncologist at least every 3 to 4 months for the first year and every 6 months for the next 5 years.

Total serum PSA measurements are obtained during follow-up visits per standard-of-care. Biochemical progression is defined by PSA ≥ 0.2 ng/mL and rising after completion of SRT (second confirmatory value must be rising and separated by ≥ 1 month). Additional labs are drawn per the discretion of the treating physician.

Imaging studies are done at the discretion of the treating physicians. Imaging is typically initiated at the time of suspected biochemical or clinical recurrence, and may include CT, MRI, or PET. The treating physician decides if biopsy confirmation is necessary or not.

UCLA Nuclear Medicine Research investigators or their staff will conduct telephone follow-up with enrolled patients at 3–4-month intervals for the first year and then at 6-month intervals. The investigators or staff will ask the patient for their most recent PSA value and the draw date, if and when any additional salvage therapy has been initiated, and if and when any imaging studies suggest radiographic progression. Research investigators or their staff may also conduct telephone or secure email follow-up with the treating physicians to identify changes to the initial general treatment plan (prostate bed alone, prostate bed with ADT, prostate bed and nodes, prostate bed and nodes with ADT). The study team may request more details about the actual radiation treatment plan at the discretion of the patient and the treating physician. Although we acknowledge that toxicity assessments, both patient reported and physician reported, are valuable components of prospective trials, the design of the trial makes rigorous assessments of these difficult.

#### Study duration

We expect to enroll the 193 patients within 2 years of study initiation. Patients will be followed by UCLA Nuclear medicine (phone calls/ secure emails) until either one of the following conditions occur:

1. 5 years after the date of initiation of randomization.
2. Biochemical progression.
3. Diagnostic of extra-pelvic metastatic disease by any imaging or biopsy.
4. Initiation of any additional salvage therapy.
5. Death.

### Sample size determination

In our a previous study [38], 52/270 patients (19%) had at least one lesion detected by PSMA PET/CT which was not covered by the standard radiation field that covered both the prostate bed and pelvic lymph nodes (RTOG consensus delineations). Standard SRT would not have resulted in durable disease control because gross disease would have been missed. Therefore, we hypothesized that the incorporation of PSMA PET/CT to SRT planning will improve 5-year PFS survival by 20%. Based on available published literature we estimated the 5 y PFS at 60% with standard SRT [5–9, 12]. Therefore, we assume 5-year PFS to be 60% in Arm 1 (standard SRT) and 80% in Arm 2 (PSMA PET/CT based SRT). We also assume that approximately 13% of subjects randomized to Arm 2 will have extra-pelvic metastasis detected by PSMA PET/CT, and therefore are not curable by SRT directed to the pelvis [38]. Based on these estimates, 193 subjects are required to be randomized in a 1:1.13 ratio (90 in control group and 103 in the PSMA group). Randomized, eligible, sample size in each group is therefore *n* = 90. When the randomized, eligible, sample size in each group is 90, with an estimated total number of events required of 46, an exponential maximum likelihood test of equality of survival curves with a 0.050 two-sided significance level will have 80% power to detect the difference between a PSMA group exponential parameter of 0.0446 (assuming a 5 year failure rate of 20%) and a control group exponential parameter of 0.1022 (assuming a 5-year failure rate of 40%), which equals a constant hazard ratio of 0.436; this assumes a maximum follow-up time of 5 years and a common exponential dropout rate of 0.0211 (assuming 10% 5-year drop-out rate in each group). The planned log-rank test should have similar power to the exponential MLE survival test.

### Allocation sequence generation, concealment mechanism and implementation

UCLA Department of Medicine Statistics Core (DOMStat) will build code to randomly assign patients to control/PSMA groups after the patient eligibility form is filled out in Research Electronic Data Capture (REDCap) [51]. DOMStat will develop reproducible code to randomly generate the allocation sequence. To ensure balance between treatment allocation throughout the study, we will use a blocked randomization of size 6. This block size will be unknown to the nuclear medicine research team and the radiation oncologists when enrolling a patient and the control/PSMA allocation will be masked until after the until after screening/baseline data are entered and filled out in REDCap (no anticipation of the group assignment possible). All the data management such as the randomization allocation will be performed by UCLA Nuclear Medicine Research Team in the REDCap online database.

This is an open label study. Trial participants, care providers, outcome assessors, and data analysts will be aware of the assignment after enrollment in REDCap is completed. The randomization number and assignation will be communicated 1 day after the registration by phone or email to the treating physician and the patient.

### Data collection, management and monitoring

Study database will be developed by DOMStat using REDCap [51], which is supported by the UCLA CTSI program and includes high level data security, access logs, data storage and backup. DOMStat has an extensive computational infrastructure with database and statistical software, desktop computers, and a centralized file server for data storage and backup. The REDCap study database will have validated range checks for data entry fields, branching logic, and rigorous pre-testing to make sure the data are appropriately capture. The UCLA Nuclear Medicine research team will enter all data of each patient into the REDCap database. The UCLA Nuclear Medicine research team will have full access to all interim and final results of the study through the REDCap database and is responsible for the final decision to terminate the trial. There is no planned interim analysis. All the data management will be performed by the UCLA Nuclear Medicine Research Team in the REDCap online database. During the clinical investigation, the UCLA Nuclear Medicine research team will evaluate the progress of the trial, including periodic assessments of data quality and timeliness, participant recruitment, accrual and retention, participant risk versus benefit, and other factors that can affect study outcome. All the datasets generated during the current study will be stored and managed on the UCLA REDCap database. All data generated and/or analyzed during this study will be publicly available (own DOI) after completion of the study and the publication of the article of the final analysis of study. The datasets generated and/or analyzed during the trial will not be publicly available before completion of the study but can be available from the corresponding author on reasonable request. Even if the required number of patients to reach statistical power (*n* = 193) is not met, patients already enrolled in the trial will still be followed for 5 years as this data alone would be valuable and unique.

### Statistical methods

We will use a log rank test to compare PFS time between the two randomized treatment arms. We assume that approximately 13% of subjects randomized to Arm 2 will be found to be ineligible for SRT and will not be included for the primary endpoint analysis. Secondary analyses will utilize Cox-proportional hazards regression models. These models will include terms for treatment as well as appropriate clinical/demographic covariates (e.g., ADT, pelvic LN RT, PSA doubling time, Gleason grade, T stage, age, etc.). Residual analyses will be performed to evaluate the proportional hazards assumptions of the Cox model. As a sensitivity analysis, we will also consider survival models that can account for competing risks (ex. death from other causes).

## Discussion

^68^Ga-PSMA-11 PET/CT molecular imaging is highly sensitive to detect and localize PCa BCR. However, it is unclear if incorporation of PSMA PET/CT imaging into the planning of SRT could improve its likelihood of success. No randomized prospective trial has been designed to determine whether PSMA PET/CT can improve 5-year outcomes in patients with early BCR after radical prostatectomy**.** The purpose of this trial is to compare the success rate of SRT in patients with BCR after radical prostatectomy among patients with PSMA PET/CT based SRT planning vs. standard SRT planning.

Potential pitfalls in study design include i) drop-out of patients randomized to the control arm as patients may be able to undergo PSMA PET/CT scans in other institutions; ii) potential FDA approval of PSMA PET imaging probes (Gallium-68-PSMA-11 or Fluor-18-DCFPyL) in the near future which would in essence lead to termination of new enrollment. As PSMA PET/CT imaging may become standard-of-care, randomizing patients to the control arm would no longer be feasible. Therefore, the time period for patient recruitment may be limited (1 to 2 years starting from September 2018). Even if the required number of patients to reach statistical power (*n* = 193) is not met, patients already enrolled in the trial will still be followed for 5 years as this would remain highly valuable and unique data.

Published randomized prospective trials with long-term follow-up demonstrate that the success rate of SRT is enhanced by the addition of concurrent and adjuvant conventional androgen deprivation [6] or first generation antiandrogens [52]. Ongoing trials are now evaluating the role of second-generation systemic therapies that target the Androgen Receptor (NRG GU 006). The magnitude of the impact of adding these systemic therapies to SRT depends on clinicopathologic features including pre-SRT PSA, Gleason Grade, margin status, and genomic classifiers [53]. Moreover, hormonal agents have well known and expected side effects. As such, the expected benefit of adding systemic therapy to SRT may be outweighed by known risks for many patients.

Oligometastatic prostate cancer, variously defined as metastatic disease with between three to five sites of identifiable metastases, is another disease state with rapidly evolving treatment paradigms. STAMPEDE randomized patients with metastatic hormone sensitive prostate cancer to long-term androgen suppression with or without radiotherapy directed to the prostate alone [54]. Pre-planned analyses of patients with limited metastatic disease burden had an improvement in survival [54]. The value of controlling recurrent local disease in patients who have synchronous metastatic prostate cancer is unknown. However, the identification of these patients with modern imaging, such as PSMA PET/CT, will increase. Indeed, we anticipate that 13% of patients randomized to PSMA PET/CT in our trial may fall into this group [38]. The optimal treatment for these patients remains unknown.

This is the first prospective randomized phase 3 trial designed to determine whether a molecular imaging modality, PSMA PET/CT, can improve outcomes after SRT. Like testing the addition of systemic therapies to SRT, testing the addition of PSMA PET/CT to SRT may improve disease control. However, unlike additional systemic therapies, PSMA PET/CT has few if any side effects, minimal risks, and enables better patient selection and disease state identification.

## Abbreviations

68Ga: Gallium-68
ADT: Androgen deprivation therapy
BCR: Biochemical recurrence
BPFS: Biochemical progression-free survival
CT: Computed tomography
CTV: Clinical Target Volume
DICOM: Digital imaging and communications in medicine
FDA: Food and Drug Administration
GBq: GigaBecquerel
GCP: Good Clinical Practice
HIPAA: Health Information Portability and Accountability Act
IND: Investigational new drug
IRB: Institutional review board
MBq: MegaBequerel
mCi: Millicurie
MRI: Magnetic resonance imaging
NCCN: National comprehensive cancer network
NDA: New drug application
PCa: Prostate cancer
PET/CT: Positron emission tomography/computed tomography
PFS: Progression-free survival
PSA: Prostate-specific antigen
PSMA: Prostate-specific membrane antigen
REDCap: Research Electronic Data Capture
RTOG: Radiation Therapy Oncology Group
SBRT: Stereotactic Body Radiation Therapy
SRT: Salvage Radiation Therapy
UCLA: University of California, Los Angeles
US: United States
