# Correction to: Randomized prospective phase III trial of 68Ga-PSMA-11 PET/CT molecular imaging for prostate cancer salvage radiotherapy planning [PSMA-SRT]

**DOI:** 10.1186/s12885-019-5297-x

**Published:** 2019-01-21

**Authors:** Jeremie Calais, Johannes Czernin, Wolfgang P. Fendler, David Elashoff, Nicholas G. Nickols

**Affiliations:** 1 Department of Molecular & Medical Pharmacology, Ahmanson Translational Theranostics/ Imaging Division, University of California, Los Angeles, USA; 2 Department of Nuclear Medicine, University Hospital Essen, Essen, Germany; 3 Department of Medicine Statistics Core (DOMStat), UCLA CTSI Biostatistics and Computational Biology, University of California, Los Angeles, USA; 4 Department of Radiation Oncology, University of California, Los Angeles, USA; 5 Department of Radiation Oncology, VA Greater Los Angeles Healthcare System, Los Angeles, CA USA

**Correction to: BMC Cancer**

**https://doi.org/10.1186/s12885-018-5200-1**

Following publication of the original article [1], we have been notified that one of the author names was listed incorrectly. Both incorrect and correct author names are presented below. The original publication has been corrected.

- Originally published name:
  - Nicholas Nicholas G. Nickols
- Correct name:
  - Nicholas G. Nickols
